# PROTOCOL: Effectiveness of social accountability interventions in low‐ and middle‐income countries: An evidence and gap map

**DOI:** 10.1002/cl2.1430

**Published:** 2024-11-05

**Authors:** Mirza Hassan, Howard White, Iffat Zahan, Ashrita Saran, Shamael Ahmed, Semab Rahman, Shabnaz Zubaid

**Affiliations:** ^1^ BRAC Institute of Governance and Development (BIGD) BRAC University Dhaka Bangladesh; ^2^ Campbell South Asia New Delhi India

**Keywords:** governance, intervention, LMIC, outcome, social accountability

## Abstract

This is the protocol for an evidence and gap map, which aims to map the existing evidence on the effectiveness of social accountability interventions in low‐ and middle‐income countries. This map will help users identify the size and quality of the evidence base, guide strategic program development, and highlight gaps for future research. The map will cover studies published after 2000, including systematic reviews, randomized controlled trials, non‐experimental designs, and before‐after designs.

## 1 BACKGROUND

### 1.1 The problem, condition or issue

A study conducted by the Institute of Development Studies (IDS) in 2010 to research the “Impact of Social Accountability” found that almost no “meta literature” on accountability impact and effectiveness was present, and the existing literature was variable and scattered (McGee & Gaventa, 2011). Moreover, the existing literature was largely theoretical with under‐specified assumptions with very few studies of the impact and effectiveness in real terms (Boydell & Keesbury, 2014). Social accountability (SA) is an advancing umbrella category that includes citizen monitoring and oversight of public and/or private sector performance, user‐centered public information access/dissemination systems, public complaint and grievance redress mechanisms, as well as citizen participation in actual resource allocation decision‐making (Fox, 2015).

**Chart 1:**
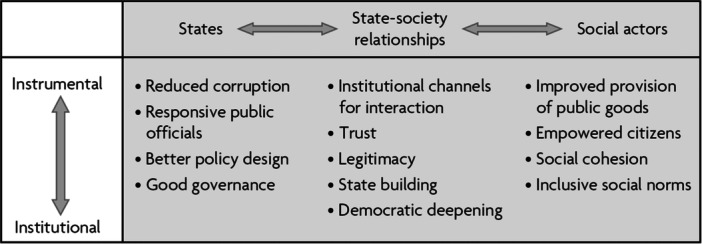
Adopted from Grandvoinnet et al. (2015).

Joshi (2017) explained SA “as a subset of accountability more broadly comprises … citizens' efforts at ongoing meaningful collective engagement with public institutions for accountability in the provision of public goods.” Lodenstein et al. (2013) mentioned that “social accountability (also called citizen‐driven accountability or bottom‐up accountability) refers to the strategies, processes or interventions whereby citizens voice their views on the quality of services or the performance of service providers or policymakers who, in turn, are asked to respond to citizens and account for their actions and decisions.” The paper emphasized that the relevance of SA can be described from two perspectives. According to “Institutional economic perspective,” SA works as “complementary” to the administration that emphasizes on the “regulatory mechanism of monitoring.” However, besides this, SA can be described from broader trends in development, such as “participatory governance.” Based on these perspectives, the systematic review listed the expected outcomes of the SA initiatives to be “reduction in corruption; better governance and policy design; enhanced voice, empowerment and citizenship of marginalized groups; responsiveness of service providers and policymakers to citizens' demands and, ultimately, the achievement of rights, health and developmental outcomes” (Lodenstein et al., 2013). Grandvoinnet et al. (2015) presented a chart to show the “expanded impact” of SA (Chart 1). The author argued that it is difficult to conclude whether SA works or not. One of the main reasons is complexity of potential outcomes from SA process. The paper includes a wide range of important outcomes, such as “benefits in the state (better governance), in state‐society relationships (increased legitimacy), and in society (improved provision of public goods) as well as instrumental benefits (improved provision of public goods) and institutional benefits (state building)” (Grandvoinnet et al., 2015). Marston et al. (2020) in their systematic review mentioned that SA interventions may ensure better service delivery by providing for example “better delivery of services (e.g., via citizen report cards, community monitoring of services, social audits, public expenditure tracking surveys, and community‐based freedom of information strategies); better budget utilization (e.g., via public expenditure tracking surveys, complaint mechanisms, participatory budgeting, budget monitoring, budget advocacy, and aid transparency initiatives); improved governance outcomes (e.g., via community scorecards, freedom of information, World Bank Inspection Panels, and Extractives Industries Transparency Initiatives); and more effective community involvement and empowerment (e.g., via right to information campaigns/initiatives, and aid accountability mechanisms that emphasize accountability to beneficiaries).” With the development of the concept of SA over the decades, new outcomes such as community empowerment, providers' responsiveness are at the heart of discussions too (Hamal et al., 2018).

We can see that SA measures can go a long way in helping the poor, vulnerable and marginalized groups of society. In Asian countries, the marginalized represents a majority of the society and research suggests that compared to other regions, SA initiatives across Asia, particularly the south and south‐east, have a much greater element of community participation and involvement (World Bank, 2007). Likewise, in many low‐ and middle‐income countries (LMICs), the engagement between civil society groups and governments in many of these initiatives is very prominent in comparison to that in other developed regions. Also, focusing on LMICs would help to maintain consistency because SA mechanisms tend to be contextual specific. There is a growing recognition that context is critical in shaping, making, and breaking SA interventions (O'Meally, 2013).

Recent years have witnessed growing concerns about issues of governance and accountability in developing countries. There is an emergent need for identifying and promoting approaches towards building accountability that rely on civic engagement. SA includes an ever‐widening range of concepts and distinct practices that needs to be identified, analyzed and researched upon in depth.

SA is about including citizens and communities within the forms of administration so that choices and activities of the individuals and associations with control are made open and can be addressed. To respond to the developing requirement for accountability as well as transparency, numerous multi‐stakeholder activities have been made to reinforce public information revelation. These activities create a platform point to empower the society organizations to utilize the data and engage with the government for better development outcomes. These practices are initiated by a wide variety of factors including government agencies, different community platforms, political leaders, and aid and donor agencies. They use different forms of informal and formal strategies via support for formal and informal groups or organizations.

### 1.2 The intervention

The main objective is to map the size of the evidence of the SA interventions in LMICs. There can be multiple interventions through which SA outcomes can be achieved. The dimensions of this evidence and gap map (EGM) will place interventions in rows and outcomes in columns. The interventions related to access to information, citizens' voice and representation, governance, mobilization actions, resource monitoring actions will be listed. The column will be arranged by outcomes related to performance improvement of public and private institutions, service quality, allocative efficiency, citizen engagement, inclusion, responsiveness, quality of life, knowledge and learning outcomes from this project. This map outline is elaborately discussed in the EGM framework section later on.

The map will summarize the SA interventions in LMICs. We will map the studies published after the Year 2000. The map will accommodate systematic reviews, randomized control trials, non‐experimental design and before‐after design.

### 1.3 Why it is important to develop the EGM

An evidence gap map on the effectiveness of SA interventions in LMICs will help users to identify the size and quality of the evidence base of SA interventions in these countries; guide users to available high quality evidence to inform strategy and program development; show users where there is no high quality evidence; identify gaps to be filled by evidence synthesis and new studies for researchers and research commissioners – thus, providing a more strategic, policy‐oriented approach to research agenda. This approach will assist in effective result based decision making and action oriented planning.

There is one related map on state society relations which consolidates evidence on interventions to improve state‐society relations in LMICs (Phillips et al., 2017), however the focus is different from ours. There are other EGMs on SA interventions specific to different sectors (Rathinam et al., 2019; Munar et al., 2018). As far as we know, there are no EGMs that address the objectives that we have.

## 2 OBJECTIVES

The objective of this EGM will be to answer the following questions:

- What is the size and quality of the evidence base of SA interventions in LMICs?
- Where is the high‐quality evidence which can be used to inform strategy and program development?
- Where are the gaps in evidence that can be filled in with new studies and interventions?

## 3 METHODS

### 3.1 EGM: Definition and purpose

Mapping the evidence in an existing area has been used since the early 2000s (Saran & White, 2018). An EGM, being a systematic evidence synthesis by nature requires some systematic steps to follow. EGM primarily identifies the evidence gap in a selected field that can be filled by new evidence; thus, making it easier for experts to introduce new evidence in their given field by looking at the map. It also allows existing evidence to be easily discovered by researchers, policy makers and “research commissioners” (White et al., 2020). To map high quality evidence and measure the size and quality of the evidence base of SA interventions in LMICs, we aim to develop this map following stages like Welch et al. (2021):

- Define a framework with stakeholder engagement
- Identify the existing evidence using a pre‐defined search strategy
- Appraise the quality of the evidence
- Analyze the data to measure the size of the evidence and identify the gap
- Present the findings and map in a user‐friendly manner

### 3.2 Framework development and scope

Our EGM will focus on the wide range of interventions on SA. We will use a well‐known conceptual framework to define our intervention and outcome categories. To do this, we will use existing literature and consult with subject experts to develop a concrete framework. The field of SA is growing rapidly, hence we will ensure our framework captures new interventions and outcomes along with traditional ones. To generate the framework, we will follow the following steps:

- *Brainstorming workshop*: In this workshop, the research team has identified possible intervention and outcome categories.
- *Stakeholder engagement*: We have then shared the basic frameworks with the subject experts for their input.
- *Testing the framework*: After revising the framework according to the suggestions of the subject experts, we developed a coding exercise and tested the framework with 10 studies.
- *Developing tools*: After revising the framework again while testing, we developed a coding tool, search strategy and eligibility criteria.

### 3.3 Stakeholder engagement

We have developed a team consisting of experts within BIGD, along with the researchers. The researchers developed a draft framework in consultation with internal expert. The draft was then presented to a team at Global Partnership for Social Accountability (GPSA). After multiple consultations with GPSA and Campbell South Asia, the framework has been finalized. The framework was tested through pilot coding of 20 studies. Although the core research team does not include an information specialist, the search strategy has been reviewed by the information specialist from Campbell South Asia. The team also presented the EGM framework at the Global evidence summit, 2020 and gathered feedback. The stakeholders will be engaged at the time of report finalization and dissemination.

### 3.4 Conceptual framework

**Figure 1:**
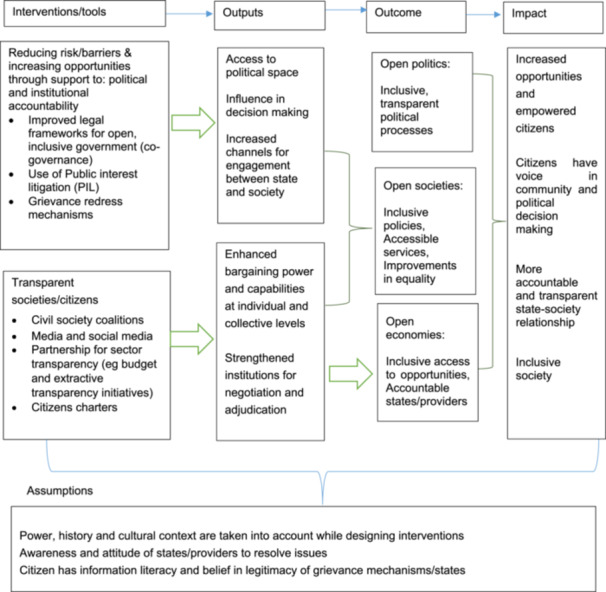
Theory of change for social accountability interventions. *Source*: Itad (2016), Franco & Shankland (2018), Joshi (2014).

The concept of SA is continuously evolving. The concept involved a wide range of interventions that builds citizens' as well as the state's power. It includes different categories such as access to information, citizen's voice and representation, governance, mobilization actions, and resource monitoring actions (Table 1). The interventions are intended to bring about different outcomes (Table 2) such as performance improvement of public and private actors/institutions, service quality, allocative efficiency, citizen engagement, inclusion, responsiveness, quality of life, knowledge and learning outcomes from this project. Thus, this map will be built on a combination of frameworks of different interventions. Figure 1 shows a causal line from interventions to impact for SA interventions. This has been developed based on the causal map and literature by Itad (2016), Franco & Shankland (2018), Joshi (2014) and Fox (2015).

**Table 1:** Intervention categories and sub‐categories.

	Intervention	Intervention sub‐category
1	Access to information	Information sharing through media
		Information sharing to targeted audience
		Service/citizen charters
		Freedom of information initiative/Right to information
2	Citizens voice and representation	Forums/town hall meetings
		Capacity building of Civil society groups/organizations (CSO)/CBOs
		Community score cards/Citizens report cards/social audits/citizen feedback
		Social Movements
		Use of Public interest litigation (PIL)
		Grievance redress mechanisms
3	Governance	Local agencies and committees
		Co‐governance
		Networked Governance
4	Mobilization Actions	Information Campaigns
		Social mobilization campaigns
		Participation in PTA
5	Resource Monitoring actions	Resource Allocation and Management
		Participatory budget formulation
		Participatory budget analysis
		Public expenditure tracking surveys (PETS)
		Monitoring contracting and procurement processes

**Table 2:** Outcome categories and sub‐categories.

	Outcome	Outcome sub‐category
1	Performance improvement of public and private actors/institutions	Increased Transparency
		Reduced Corruption
		Public confidence or trust in politicians or institutions
		Quality/effectiveness of government and institutions
		Answerability
2	Service Quality	Efficiency of service delivery
		Service Utilization
		Service provider absenteeism
		Citizen/user satisfaction
3	Allocative Efficiency	Budget Formulation
		Budget Utilization
		Resource Allocation
4	Citizen Engagement	State‐society relations/government‐citizen linkages
		Channels for engagement and negotiation
		Meetings and forums
		Participation/attendance
5	Inclusion	Targeting efficiency in program design
		Marginal group representation in policy formulation
		Marginal group oversight in policy implementation
6	Responsiveness	Responsiveness of service providers to citizen's needs/demand)
		Grievance redressed
		Policy Influence/Changes
7	Quality of life	Overall well‐being and Quality of life
		Health outcomes
		Social outcome
		Education outcomes
		Economic empowerment
8	Knowledge and Learning Outcomes from Project	Refined SA interventions
		Increased sustainability
		Replication or scaling up

## 4 DIMENSIONS

### 4.1 Types of study design

This EGM will focus on the effectiveness of SA intervention. This will include all effectiveness studies and systematic reviews. Studies that follow the below designs will be included:

- *Systematic review*: Review of primary studies adopting systematic approach, which includes: (1) clear inclusion and exclusion criterion, (2) an explicit search strategy, (3) a systematic coding and analysis of included studies, (4) meta‐analysis (where possible)
- *Randomized Control Trial*: Random assignment of the intervention, including natural experiments
- *Non‐experimental design with comparison group*: Non‐experimental studies with comparison group including regression‐based designs (DD, RDD, IV, Matching, cohort, etc.)
- *Before versus after design*: Pre‐post outcome measurement with no comparison group
- We don't plan to include any qualitative research.

### 4.2 Types of intervention/problem

The EGM will focus on five broad categories of intervention. Within each of these categories, there will be different sub‐categories (Table 1). The definitions can be found in Supporting Information: Additional Table A1 at the end.

- Access to information
- Citizens voice and representation
- Governance
- Mobilization Actions
- Resource Monitoring action

### 4.3 Types of population (as applicable)

This EGM will focus on the intervention of SA in both rural and urban areas of LMICs. The following population will be included:

- Women and girls,
- Elderly,
- Children
- People with history of mental illness
- People with Complex Needs/Dual Diagnosis
- Adults/youths
- LGBT community
- Survivors of Domestic Violence/Abuse
- People with Disabilities
- Migrants
- Indigenous people

### 4.4 Types of outcome measures (as applicable)

This EGM will focus on 8 categories of outcome (Table 2): (The definitions can be found at Supporting Information: Additional Table A2).

- Performance improvement of public and private actors/institutions
- Service Quality
- Allocative Efficiency
- Citizen Engagement
- Inclusion
- Responsiveness
- Quality of life
- Knowledge and Learning Outcomes from Project

### 4.5 Types of location/situation (as applicable)

Our EGM location is LMICs.

### 4.6 Types of settings (as applicable)

Studies in the following settings will be included and excluded:

#### Inclusion criteria

- Studies that include primary and systematic reviews of the effectiveness of SA.
- All the studies after the year 2000.
- Experimental studies, pre‐post outcome measurement, non‐experimental design with or without comparison group.
- Studies which focus on LMICs.
- Studies that are either completed or ongoing.
- Journal articles, as well as gray literature.

#### Exclusion criteria

- Studies that are not focusing on LMICs
- Studies that do not include any of the inclusion criteria.
- Studies before the year of 2000
- Studies that are qualitative

These criteria are provided in detail in the Supporting Information S1: Additional Table A3. Screening tool for including and excluding studies is in Supporting Information S1: Table A4 of the same section.

## 5 SEARCH METHODS AND SOURCES

The search will be as comprehensive as possible, using (but not limited to) relevant bibliographic databases, web‐based search engines, websites of specialist organizations, bibliographies of relevant reviews, and targeted calls for evidence using professional networks or public calls for submission of articles.

The following databases will be searched electronically for this EGM:

1. International Organizations
  - Who Global Library

2. Academic Databases:
  - JSTOR
  - Scopus
  - The National Bureau of Economic Research (NBER)
  - Social Science Research Network (SSRN)
  - Emerald Insight
  - Taylor & Francis
  - Google Scholar

3. Bibliographic Databases:
  - PubMed via National Library of Medicine
  - International Bibliography of Social Sciences (IBSS)
  - Applied Social Sciences Index and Abstracts (ASSIA) via ProQuest
  - Web of Science Master Journal List via Clarivate
  - IDEAS via RePEc (Research Papers in Economics)

4. Systematic Review Database:
  - 3ie Systematic Review Databases
  - Campbell Collaboration
  - Cochrane Database of Systematic Reviews

5. LMIC – specific Databases:
  - Latin American and Caribbean Health Sciences and Literature (LILACs) via Virtual Health Library

6. Gray Literature search/websites
  - Abdul Latif Jameel Poverty Action Lab (J‐PAL)
  - Action Aid
  - British Library for Development Studies
  - CARE
  - Concern Worldwide Division for Social Policy & Development
  - Child Fund International
  - Gates Foundation
  - GreyNet International
  - Innovations for Poverty Action (IPA) Database
  - International Center for Research on Women (ICRW)
  - International Food Policy Research Institute (IFPRI)
  - International Rescue Committee (IRC)
  - International Red Cross
  - IPC‐IG (Working papers)
  - LLC/Center for Human Services
  - London School of Hygiene & Tropical Medicine (LSHTM)
  - MedCarib
  - Medecins Sans Frontières
  - Oak Foundation
  - One International
  - Opengrey
  - Project Concern
  - RAND Corporation
  - UN Economic and Social Council UNESCO
  - UNICEF Innocenti Research Center
  - University Research Co.
  - United Nations Population Fund
  - World Bank Group (WBG)
  - World Food Program
  - World for World Organization
  - World Vision.

Search queries are included in Appendix 1.

An example database search is included in Appendix 2.

## 6 ANALYSIS AND PRESENTATION

### 6.1 Report structure

Our EGM report will include the standard sections: executive summary, background, methods, results, and a conclusion. We will indicate any changes we made between the protocol and the final report. The executive summary will contain the summary and findings of the report. The background will describe the SA literatures and provide a brief description of how SA interventions have been evolving. We will also present a theory of change, followed by the scope of this particular EGM. This sub‐section will have description on our framework, interventions categories and outcome categories.

The report will also present a detailed search strategy, methods of including or excluding studies and how exactly the data is extracted from the included studies. A comprehensive quality appraisal plan will also be included. This section will contain a PRISMA flow chart, followed by a full search strategy for some databases in the appendix.

The results section will present the number of studies retrieved from our database search and provide an overview of the types of studies by intervention, outcomes, auspices and other filters used. The conclusion will provide a detailed account of policy implications for researchers and decision‐makers and highlight key areas for research commissioning.

We will have some main figures and tables such as:

- Conceptual framework/theory of change
- PRISMA flow chart
- Number of studies by intervention sub‐categories
- Number of studies by region

### 6.2 Filters for presentation

The unit of analyses will be number studies that are included under each sub‐category combination of intervention and outcome. We will present the EGM in a standard format: total number of studies, broken down into sub‐categories of intervention and outcome. We will also present a narrative report using figures, tables to narrate the whole process, summaries of number of included studies and coding process. The report will also discuss the limitations of the map while discussing the use of the EGM. The key dimensions will be interventions (presented in rows) and outcomes (presented in columns). We will use bubbles of varying sizes to present included studies. Different colors will be used for different types of studies. Final decisions about the filters will be made based on the number of included studies and coded information. The online interactive map will be hosted by Campbell.

### 6.3 Dependency

We will treat multiple reports (e.g., secondary analyses or protocols) of a single study as one. Systematic reviews will likely include primary studies that are included in the map, and there may be more than one systematic review which may include the same primary study. Primary studies which meet our eligibility criteria will be included in the map regardless of whether they are included in a systematic review.

## 7 DATA COLLECTION AND ANALYSIS

### 7.1 Screening and study selection

A team of two independent researchers will be responsible for searching and screening the studies initially. Any disagreement on particular studies will be resolved by the third researchers.

### 7.2 Data extraction and management

All the authors will be involved in data extraction, coding and management. Three co‐authors will independently code all eligible studies and reviews. Any disagreement will be resolved by the lead authors. Besides the “interventions” and “outcomes” mentioned in previous section, we will code several other factors to filter the studies in EGM (Table 3).

**Table 3:** Filter code for included studies.

		Definitions
1	Study design	
1.1	Systematic review	Review of primary studies adopting systematic approach, which includes: (1) clear inclusion and exclusion criterion, (2) an explicit search strategy, (3) a systematic coding and analysis of included studies, (4) meta‐analysis (where possible)
1.2	RCT	Random assignment of the intervention, including natural experiments
1.3	Non‐experimental design with comparison group	Non‐experimental studies with comparison group including regression‐based designs (DD, RDD, IV, Matching, cohort, etc…)
1.4	Before versus after design	Pre‐post outcome measurement with no comparison group
2	Publication status	
2.1	Completed	Study findings are available in a published report or paper (Published does not mean in a journal but in any form of a complete paper, NOT conference abstract or ppt).
2.2	Ongoing	The research is still in progress. There is no published paper or report of study findings.
3	Study region	
4	National Region	
4.1	Country	LMICs
		Angola, Bangladesh, Bhutan, Bolivia, Cabo Verde, Cambodia, Cameroon, Comoros, Congo, Rep., Côte d'Ivoire, Djibouti, Egypt. Arab, Rep., El Salvador, Eswatini, Ghana, Honduras, India, Indonesia, Kenya, Kiribati, Kyrgyz Republic, Lao PDR, Lesotho, Mauritania, Micronesia, Fed. Sts., Moldova, Mongolia, Morocco, Myanmar, Nicaragua, Nigeria, Pakistan, Papua, New Guinea, Philippines, São Tomé and Principe, Senegal, Solomon Islands, Sudan, Timor‐Leste, Tunisia, Ukraine, Uzbekistan, Vanuatu, Vietnam, West Bank and Gaza, Zambia, Zimbabwe and others (will be listed from World bank's website: https://datatopics.worldbank.org/world-development-indicators/the-world-by-income-and-region.html)
4.2	City	
5	Population	
5.1	Adults/youth	
5.2	Children	
5.3	Rural	
5.4	Urban	
5.5	Women and Girls	Studies where the principle population group being studied are women/girls.
5.6	Elderly	Studies where the principle population group being studied are elderly (i.e., aged 65+).
5.7	People with/History of Mental Illness	Studies where the principle population group being studied are people with (or a history of) mental illness.
5.8	People with Complex Needs/Dual Diagnosis	Studies where the principle population group being studied are people with complex needs/dual diagnosis
5.9	LGBT community	Studies where the principle population group being studied identify as LGBT
5.10	Survivors of Domestic Violence/Abuse	Studies where the principle population group being studied are survivors of domestic violence/abuse
5.11	People with Disabilities	Studies where the principle population group being studied are people with disabilities
5.12	Migrants	Studies where the principle population group being studied are migrants
5.13	Indigenous people	Studies where the principle population group being studied are ethnic groups who are the original inhabitants of a given region (first peoples, aboriginal peoples or native peoples).

### 7.3 Tools for assessing risk of bias/study quality of included reviews

We will appraise the methodological quality of systematic reviews with (1) clear inclusion and exclusion criterion, (2) an explicit search strategy, (3) a systematic coding and analysis of included studies, (4) meta‐analysis (where possible) in duplicate for 20% of eligible studies. If the agreement is over 80%, we will proceed with single data extraction with verification by a second reviewer for the remainder of studies. The methodological quality of primary studies is not usually assessed in EGMs.

### 7.4 Methods for mapping

EPPI reviewer 4 is used to perform screening and coding and we will be using the EPPI mapper utility to develop the online map.

## CONTRIBUTIONS OF AUTHORS

The recommended optimal EGM team composition includes at least one person who has content expertise, at least one person who has methodological expertise and at least one person who has statistical expertise. It is also recommended to have one person with information retrieval expertise.

- Content: Dr. Mirza HasaaPROTOCOL: n
- EGM methods: Howard White
- Statistical analysis: Iffat Zahan, Ashrita Saran
- Information retrieval: Iffat Zahan, Semab Rahman, Shamael Ahmed, Shabnaz Zubaid

## DECLARATIONS OF INTEREST

No conflicts of interest exists.

### Plans for updating the EGM

Ashrita Saran and Iffat Zahan will be responsible to update the EGM provided availability of funds.

## SOURCES OF SUPPORT

### Internal sources

- Research, Policy, and Governance (RPG) program, BIGD, BRAC University, Bangladesh

RPG is an internal program of BIGD. This evidence and gap map is funded by this program.

### External sources

- Campbell Collaboration, India

Campbell collaboration is supporting BIGD intellectually to develop this EGM.

## Supporting information

Supporting information.
