# Supplementary material for: PROTOCOL: Effectiveness of social accountability interventions in low‐ and middle‐income countries: An evidence and gap map
Source: Campbell Syst Rev. 2024 Nov 5;20(4):e1430. doi: 10.1002/cl2.1430 (PMC11538311; doi:10.1002/cl2.1430)
Supplement: Supplementary file 1 — Supporting information. [file CL2-20-e1430-s001.docx]

# Appendices

## Appendix1: Key words for search queries

| Key concepts | Free text terms |  |
| --- | --- | --- |
| LMIC keywords | Developing nations, third world, low income, poor, underdeveloped  Middle income, emerging economies  Asia, Africa, Latin America, Europe, conflict |  |
| Population key words | Citizens, community, tribe, people, residents  Adult, elderly  Women, girls  Youth, young adults  Children, infants  Rural, village, suburb  Urban, city, town  District, state, division |  |
| Social Accountability intervention keywords | Citizen engagement, voice, participation, representation  Mobilisation, campaign, network, movement  Access to information, freedom of information, right to information, media, online, digital  CSO, CBO, associations, agencies, NGO, informal, private  Formal, public, government, state, national  scorecard, report card, audit information, monitoring, evaluation  grievance redress, litigation, complaint, online, digital  participatory budget, resource allocation, planning |  |
| Outcome keywords | Performance, attendance, absenteeism  service quality, service delivery  quality of life, empowerment, inclusion,  transparency, access, awareness  trust, confidence  corruption, misreporting, bribe  efficiency, effectiveness, improvement  learning, experience, |  |
| Study design keywords | RCT, experiment, quantitative, lab, natural, impact evaluation,  Analysis, regression |  |
| Sector keywords | Education, school, parent teacher association, student attendance, student performance  Health, healthcare, community hospitals, health workers, mortality rate, reproductive health, disease  Agriculture  WASH  Housing  Political  Judicial  Humanitarian & disaster (COVID-19)  Poverty, inequality |  |

## Appendix2: Suggested search terminology with results

Database: JSTOR

Searched on 2nd June 2021

| # | Searches | Results |
| --- | --- | --- |
| 1 | “social accountability” | 2814 |
| 2 | "social accountability" OR "citizen engagement" OR "citizen participation" OR "citizen representation" | 24,534 |
| 3 | "social accountability" OR "citizen engagement" OR "citizen participation" OR "citizen representation" OR "citizen mobilization" OR "access to information" | 66,834 |
| 4 | 1+2+3  (“social accountability”) OR (“information dissemination”) OR (“right to information”) OR (“service charters”) OR (“citizen engagement”) OR (“social audits”) OR (“townhall meeting”) OR (“grievance mechanism”) OR (“community scorecard”) OR (“citizen report card”) OR (“public interest lawsuit”) OR (“participatory budgeting”) OR (“access to budget”) OR (“budget planning”) OR (“budget monitoring”) OR (“public expenditure tracking”) OR (“citizen mobilization”) OR (“resident welfare association”) OR (“slum dweller federation”) | Too long for JSTOR |
| 5 | “social accountability” AND ((“transparency”) OR (“corruption”) OR (“accessibility”) OR (“government citizen linkages”) OR (“trust”)) | 1595 |
| 6 | “social accountability” AND ((“service quality”) OR (“efficiency”) OR (“service utilization”) OR (“citizen satisfaction”) OR (“user satisfaction”) OR (“public service quality”)) | 826 |
| 7 | “social accountability” AND ((“empowerment”) OR (“electoral outcome”) OR (“democratic spaces”) OR (“policy influence”)) | 452 |
| 8 | “social accountability” AND ((“efficiency”) OR (“budget utilization”) OR (“resource allocation”) OR (“inclusiveness”) OR (“targeting performance”) OR (“inclusive policy”) OR (“marginal groups”)) | 945 |
| 9 | “social accountability” AND ((“responsiveness”) OR (“services to user responsiveness”) OR (“attitude of officials”) OR (“health welfare”) OR (“poverty reduction”)) | 818 |
| 10 | 5+6+7+8+9  “social accountability” AND ((“transparency”) OR (“corruption”) OR (“accessibility”) OR (“government citizen linkages”) OR (“trust”) OR (“service quality”) OR (“efficiency”) OR (“service utilization”) OR (“citizen satisfaction”) OR (“user satisfaction”) OR (“public service quality”) OR (“confidence in public service”) OR (“empowerment”) OR (“electoral outcome”) OR (“democratic spaces”) OR (“policy influence”) OR (“efficiency”) OR (“budget utilization”) OR (“resource allocation”) OR (“inclusiveness”) OR (“targeting performance”) OR (“inclusive policy”) OR (“marginal group representation”) OR (“responsiveness”) OR (“services to user responsiveness”) OR (“attitude of officials”) OR (“health welfare”) OR (“poverty reduction”)) | Too Long for JSTOR |
| 11 | “social accountability” AND “low and middle income countries” | 25 |
| 12 | “social accountability” AND ((“citizens”) OR (“youth”) OR (“young people”) OR (“rural citizens”) OR (“urban citizens”) OR (“humanitarian”) OR (“tribal”) OR (“marginalized populations”) OR (“women”)) | 1803 |
| 13 | 4+ 10 + 12  ((“social accountability”) OR (“information dissemination”) OR (“right to information”) OR (“service charters”) OR (“citizen engagement”) OR (“social audits”) OR (“townhall meeting”) OR (“grievance mechanism”) OR (“community scorecard”) OR (“citizen report card”) OR (“public interest lawsuit”) OR (“participatory budgeting”) OR (“access to budget”) OR (“budget planning”) OR (“budget monitoring”) OR (“public expenditure tracking”) OR (“citizen mobilization”) OR (“resident welfare association”) OR (“slum dweller federation”)) AND ((“transparency”) OR (“corruption”) OR (“accessibility”) OR (“government citizen linkages”) OR (“trust”) OR (“service quality”) OR (“efficiency”) OR (“service utilization”) OR (“citizen satisfaction”) OR (“user satisfaction”) OR (“public service quality”) OR (“confidence in public service”) OR (“empowerment”) OR (“electoral outcome”) OR (“democratic spaces”) OR (“policy influence”) OR (“efficiency”) OR (“budget utilization”) OR (“resource allocation”) OR (“inclusiveness”) OR (“targeting performance”) OR (“inclusive policy”) OR (“marginal group representation”) OR (“responsiveness”) OR (“services to user responsiveness”) OR (“attitude of officials”) OR (“health welfare”) OR (“poverty reduction”)) AND ((“citizens”) OR (“youth”) OR (“young people”) OR (“rural citizens”) OR (“urban citizens”) OR (“humanitarian”) OR (“tribal”) OR (“marginalized populations”) OR (“women”) OR (“adults”) OR (“elderly”)) | Too long for JSTOR |
| 14 | 5+6, limit with “citizens”  “social accountability” AND (“transparency” OR “corruption” OR “accessibility” OR “service quality” OR “citizen satisfaction” OR “government citizen linkages” OR “budget utilization”) AND “citizens” | 2268 |

Additional tables

## Table A1: Definitions of intervention categories

|  | Intervention Categories | Definitions | | Sources |
| --- | --- | --- | --- | --- |
| **1** | **Access to information** | | | |
|  | Information sharing through media | Information sharing through media where media is defined to include mass media, digital media (internet and mobile). Tools include: media reports, Investigative journalism e.g. reports by newspapers on crimes, online information database, online application for information, Posts by Internet bloggers | | UNDP (2012). Social Accountability in a Changing Region-Actors and Mechanisms |
|  | Information sharing to targeted audience | This means dissemination of information to a specific audience. It includes: public/community dissemination of information on audit, funding etc., information dissemination workshop on citizen rights, Educational trainings | | Fox, J. A. (2015). Social accountability: what does the evidence really say? World Development, 72, 346-361 |
|  | Service/citizen charters | Public document that sets out basic information on the services provided, the standards of service that customers can expect from an organisation, and how to make complaints or suggestions for improvement | | OECD, Löffer E, Parrado S, Zmeskal T (2007) Improving customer orientation through service charters. A handbook for improving quality of public services. OECD/Ministry of Interior the Czech Republic/Governance International, Paris |
|  | Freedom of information initiative/Right to information | Policy initiatives to advance new, modify or reform existing transparency and access to information legislation or regulations (national, state/provincial, municipal, sector) | | GPSA Note 2: Responsive and Multi-pronged strategies, Hazell, Robert, and Ben Worthy. "Impact of FOI on central government." Constitution Unit, University College London (2009). |
| **2** | **Citizens voice and representation** | | | |
|  | Forums/town  hall meetings | Regularly held platforms for public representatives to meet with ordinary citizens to share information or receive input. | | Tembo, F., 2013. Rethinking Social Accountability in Africa: Lessons from the Mwananchi Programme. UK AID. |
|  | Capacity building of Civil society groups/organizations (CSO)/ CBOs | Civil society refers to the space for collective action around shared interests, purposes and values, generally distinct from government and commercial for-profit actors. Civil society includes charities, development NGOs, community groups, women's organizations, faith-based organizations, professional associations, trade unions, social movements, coalitions and advocacy groups, village health committees | | WHO |
|  | Community score cards/ Citizens report cards/ social audits/citizen feedback | Performance monitoring mechanisms: Community scorecards are designed and applied by the service providers and service users, with the central objective to make decisions and develop action plans, the best terrain for the application of which is the community level. Citizen report cards are run by professional NGOs and consulting firms, are oriented principally towards providing and disseminating information on public opinion, are most effective in relatively macro settings like large cities.  A social audit usually records and interprets the experience of the clients or citizens the organizational entity is meant to service. | | Ackerman, J.M., 2005. Social Accountability in the Public Sector: A Conceptual Discussion. Social Development Papers, no. 82. The World Bank, Ahmad, R. (2008). Governance, Social Accountability and the Civil Society, JOAAG, Vol. 3. No. 1 |
|  | Social Movements | Citizens raising their voice and taking actions is fundamental to holding the authority accountable. One of the mechanisms is social movement (Transparency Initiative) Social movements facilitate changing the political costs of those in power to adopt T&A initiatives | | Transparency Initiative and World Bank |
|  | Use of Public interest litigation (PIL) | Using the mechanism of legal action for the enforcement of interest of the public to the court of law. It is a weapon by which poor, helpless or disabled seek judicial redress by filing an application to the High Court. | | Walia, I. (2009, November 20). Public Interest Litigation: An Expression of Voice for the Sufferers of Silence. |
|  | Grievance redress mechanisms | GRMs are institutions, instruments, methods, and processes by which a resolution to a grievance is sought and provided. | | Designing and Implementing Grievance Redress Mechanism.(2010). Asian Development Bank. |
| **3** | **Governance** |  | |  |
|  | Local agencies and committees | Setting up or strengthening local committees, civil society and government working together in an institutionalized oversight body to improve effectiveness of the local governance system, e.x health committees to improve health system effectiveness. | | Boydell, V., & Keesbury, J. (2014). Social accountability: What are the lessons for improving family planning and reproductive health programs? |
|  | Co-governance | Co-governance entails fostering civil society participation and simultaneously strengthening state apparatus. When social protest, movements or consultation are insufficient, the best way to tap into the energy of society is through “co-governance,” which involves inviting social actors to participate in the core activities of the state. | | Ackerman, J., 2003, ‘Co-governance for Accountability: Beyond “Exit” and “Voice”’, University of California. |
|  | Networked Governance | Network governance can be termed as a collection of persons or institutions engaged in a policy dialogue that are not accountable to the state (although the state may have initiated the formation of the network and may be involved in directing the network); interact in an environment that is open and trusting thus facilitating the free flow of views and information within and to other networks; specifically targeting a policy problem. | | Wilikilagi, V. (2009). What is Network Governance and its Implications for Public Policy Formulation? |
| **4** | **Mobilization Actions** | | | |
|  | Information Campaigns | Campaigns which aim to improve awareness of the general public of accountability of public services and public officials |  | |
|  | Social mobilization campaigns | Campaigns which aim to mobilise general public to improve accountability of public services and public officials |  | |
|  | Participation in PTA | Mobilising collaborations between teachers and parents to improve accountability in education sector. | | <https://www.worldbank.org/en/news/feature/2019/09/20/in-mongolia-parent-teacher-associations-are-improving-learning-outcomes-and-school-transparency> |
| **5** | **Resource Monitoring actions** | | | |
|  | Resource Allocation and Management | Public spending begins with the allocation of public resources for various sectors, reflective of the needs of the poor and marginalized.- involves natural resource management |  | |
|  | Participatory budget formulation | This involves direct citizen/CSO participation in budget formulation (i.e., in proposing projects and allocating funds). Participatory budget formulation is less common and usually occurs at the local level (as in over 100 municipalities in Brazil) 23 but is also theoretically applicable at higher levels. Another approach to participatory budget formation is when civil society actors prepare alternative budgets (such as South Africa’s Women’s Budget or Canada’s Alternative Federal Budget) with a view to influencing budget formulation by expressing citizen preferences- Open budget meeting | | Malena, C., Forster, R. and Singh, J., 2004. Social accountability: An introduction to the concept and emerging practice (No. 31042, p. 1). The World Bank. |
|  | Participatory budget analysis | CSOs review budgets in order to assess whether allocations match the government’s announced social commitments. This may involve analyzing the impact and implications of budget allocations, demystifying the technical content of the budget, raising awareness about budget-related issues and undertaking public education campaigns to improve budget literacy | | Malena, C., Forster, R. and Singh, J., 2004. Social accountability: An introduction to the concept and emerging practice (No. 31042, p. 1). The World Bank. |
|  | Public expenditure tracking surveys (PETS): | Monitoring resource flow: A quantitative survey of the supply side of public services that tracks the flow of public funds and material from the central government level all the way to frontline service providers | | Koziol, M., Tolmie, C., 2010. Using Public Expenditure Tracking Surveys to Monitor Projects and Small-Scale Programs: A Guidebook. The World Bank |
|  | Monitoring contracting and procurement processes | Citizens monitor and "follow the money" beyond the budget | | GPSA Note 2 |

## Table A2: Definitions of outcome categories

|  | Outcome Categories | Definitions | | Sources |
| --- | --- | --- | --- | --- |
| **1** | **Performance improvement of public and private actors/institutions** | | | |
|  | Increased Transparency | Transparency comprises the legal, political, and institutional structures that make the decision-making process and actions of public and private actors available/access to citizens - Information disclosure/Reduced information gaps - Proper reporting | | Finel in Ball, Carolyn. (2009). What Is Transparency? Public Integrity. |
|  | Reduced Corruption | Reduction of incidences of financial or administrative misreporting, investigations, prosecutions, convictions and self-reported incidences of being asked for a bribe and such measures of corruption. | | Rathinam, F., Finetti, J., Snilstveit, B., Siddiqui, Z., Chirgwin, H., Appell, R., Dickens, E. and Gaarder, M. (2019). The Effect of Transparency and Accountability Interventions in the Extractive Sectors: An Evidence Gap Map. 3ie Evidence Gap Map Report (14). New Delhi: International Initiative for Impact Evaluation (3ie). Available at: https://doi.org/10.23846/EGM014 |
|  | Public confidence or trust in politicians or institutions | Perception by the public of the performance of politicians and levels of corruption and transparency | | Rathinam, F., Finetti, J., Snilstveit, B., Siddiqui, Z., Chirgwin, H., Appell, R., Dickens, E. and Gaarder, M. (2019). The Effect of Transparency and Accountability Interventions in the Extractive Sectors: An Evidence Gap Map. 3ie Evidence Gap Map Report (14). New Delhi: International Initiative for Impact Evaluation (3ie). |
|  | Quality/effectiveness of government and institutions | Objective measures of quality and effectiveness of government | | Rathinam, F., Finetti, J., Snilstveit, B., Siddiqui, Z., Chirgwin, H., Appell, R., Dickens, E. and Gaarder, M. (2019). The Effect of Transparency and Accountability Interventions in the Extractive Sectors: An Evidence Gap Map. 3ie Evidence Gap Map Report (14). New Delhi: International Initiative for Impact Evaluation (3ie). |
|  | Answerability | The liability to sanction or reward based on the actions and performance. | |  |
| **2** | **Service Quality** | | | |
|  | Efficiency of service delivery | The choosing of means to provide a required service, with availability of sufficient material, timeliness, cost-effectiveness etc. | | Based on team discussion |
|  | Service Utilization: | It defines the means by which all planned activities will be delivered, and responsiveness in the activities will ensure uptake/coverage/outreach of services to citizens. | | Absenteeism. (n.d.). Merriam Webster. Retrieved from https://www.merriam-webster.com/dictionary/absenteeism |
|  | Service provider absenteeism | Absenteeism of teachers, health care providers, or such relevant administrators of different institutions. Absenteeism is the chronic absence from work or such institutions. | | Van Ryzin, G. G. (2004, March). The Measurement of Overall Citizen Satisfaction. Retrieved from https://www.jstor.org/stable/3381143?seq=1 |
|  | Citizen/user satisfaction | Perception of the citizens regarding the performances of the service quality and service providers with respect to the quality of basic services | | Budget, Performance and Program Analysis Handbook, Volume III. |
| **3** | **Allocative Efficiency** | | | |
|  | Budget Formulation | Budget formulation consists of all steps, actions, and documentation in the budget process that are required or that properly should be taken in advance of the enactment of an appropriation bill. It implicitly includes program performance analysis that is conducted to determine where an activity stands at present, where it is going and what alternative approaches could be taken that could better achieve objectives. The basic objectives of the formulation process are to provide a satisfactory information basis for use in making decisions about the allocation of the resources toward fulfilment of goals and needs; and to ensure that each responsible party in the process has a satisfactory opportunity to analyze the information needed to make decisions | | Kathungu, R. 2016. The Effect of Budget Utilization on the Performance of County Governments: A Case Study of Eastern Kenya Region. |
|  | Budget Utilization | Budget utilization defines the means by which all planned activities will be delivered, and responsiveness in the activities will create the outcome within the implementation period | |  |
|  | Resource Allocation | Allocation of public resources including natural resources | |  |
| **4** | **Citizen Engagement** | | | |
|  | State-society relations/ government-citizen linkages | Engagement between civil society actors and government decision makers Collaboration between social accountability initiatives of the civil society actors and the state institutions of accountability (horizontal accountability) | | GPSA Note 4: PICKING PARTNERS AND ALLIES THAT BOLSTER YOUR SOCIAL ACCOUNTABILITY EFFORTS |
|  | Channels for engagement and negotiation | Increased channel for engagement, negotiation and adjudication between the state and/or private sector and citizens - creation of new channels - efficient use of existing channels | | Rathinam, F., Finetti, J., Snilstveit, B., Siddiqui, Z., Chirgwin, H., Appell, R., Dickens, E. and Gaarder, M. (2019). The Effect of Transparency and Accountability Interventions in the Extractive Sectors: An Evidence Gap Map. 3ie Evidence Gap Map Report (14). New Delhi: International Initiative for Impact Evaluation (3ie). Available at: https://doi.org/10.23846/EGM014 |
|  | Meetings and forums | Regular use of meetings and forums to hold public actors socially accountable | |  |
|  | Participation/ attendance | Greater participation by citizens in improving social accountability of public officials | |  |
| **5** | **Inclusion** |  | |  |
|  | Targeting efficiency in program design: | Identifying the underrepresented groups (gender, sexual, ethnic, religious, socio-economic etc.) and assessing their access, capacity, limitations, opportunity costs etc. to target better. | | World Bank and ISA definitions |
|  | Marginal group representation in policy formulation | Including or seeking to include underrepresented groups (gender, sexual, ethnic, religious, socio-economic etc.) in policies to enable them to better use their skills and abilities | |  |
|  | Marginal group oversight in policy implementation | Whether marginal groups can participate in the oversight process as prescribed in the policies | |  |
| **6** | **Responsiveness** | | | |
|  | Responsiveness of service providers to citizen’s needs/demand) | Changes in behavior of service providers by taking concrete action to improve service provision in line with citizens’ concerns. For example: better queue management | | Lodenstein, E., Dieleman, M., Gerretsen, B., Broerse, J.E.W., 2017. Health provider responsiveness to social accountability initiatives in low- and middle-income countries: a realist review, Health Policy and Planning, Volume 32, Issue 1. Pages 125–140. |
|  | Grievance redressed | Investigating, verifying and responding to citizen complaints and grievance | | Based on team discussion |
|  | Policy Influence/Changes | Reforms or amendments in laws, acts etc. | | Based on team discussion |
| **7** | **Quality of life** | | | |
|  | Overall well-being and Quality of life | Improvement in overall well-being and quality of life | | Waddington, H., Sonnenfeld, A., Finetti, J., Gaarder, M., John, D., & Stevenson, J. (2019). Citizen engagement in public services in low‐and middle‐income countries: A mixed‐methods systematic review of participation, inclusion, transparency and accountability (PITA) initiatives. |
|  | Health outcomes | Nutrition status, mortality rates, physical well-being | | Rathinam, F., Finetti, J., Snilstveit, B., Siddiqui, Z., Chirgwin, H., Appell, R., Dickens, E. and Gaarder, M. (2019). The Effect of Transparency and Accountability Interventions in the Extractive Sectors: An Evidence Gap Map. 3ie Evidence Gap Map Report (14). New Delhi: International Initiative for Impact Evaluation (3ie). Available at: https://doi.org/10.23846/EGM014 |
|  | Social outcome | Measures of happiness, empowerment, gender and social relations and status, and psychological wellbeing. | |  |
|  | Education outcomes | Quality of education, there are two aspects: one is that students are not dropping out, enrolment is okay, they can enrol easily, and attendance is good. Another type of educational outcome is the quality of teaching in a classroom. |  | |
|  | Economic empowerment | This includes improvement in income, consumption, expenditure, employment, poverty and ownership, and access and rights to land | | Rathinam, F., Finetti, J., Snilstveit, B., Siddiqui, Z., Chirgwin, H., Appell, R., Dickens, E. and Gaarder, M. (2019). The Effect of Transparency and Accountability Interventions in the Extractive Sectors: An Evidence Gap Map. 3ie Evidence Gap Map Report (14). New Delhi: International Initiative for Impact Evaluation (3ie). Available at: https://doi.org/10.23846/EGM014 |
| **8** | **Knowledge and Learning Outcomes from Project** | | | |
|  | Refined SA interventions | Using learning from past or current initiatives to refine the current or future SA initiatives | | GPSA Note 5: Adaptive Learning |
|  | Increased sustainability | Using learning from past or current initiatives to increase likelihood of the sustainability of current of future SA initiatives e,g. through empowering local networks | |  |
|  | Replication or scaling up | If an initiative is a success, learning from it to replicate or scale-up. | |  |

## Table A3

##### **Type of study settings based on inclusion and exclusion criterion**

|  | Include | Exclude |
| --- | --- | --- |
| Literature type | - Published journal articles - Grey literature including technical reports which report quantitative data related to effectiveness of social accountability interventions - Technical reports/working papers | - Commentary or conceptual papers - Editorial - Conference proceedings - Qualitative studies - Case studies |
| Study Status | - Completed - Ongoing |  |
| Study design | - Studies on effectiveness - Study designs that include quantitative data - Systematic* reviews - Meta-analysis/meta regressions - Randomised controlled trial - Modelling with empirically grounded parameters/econometric studies (Regression discontinuity, propensity score or other matching techniques, difference in difference) - Instrumental variables - Other matching designs - Rigorous quasi‐experimental design/quasi‐experimental - Natural experiments - Single‐subject design - Analytical observational - Before‐after studies - Time‐series | Purely qualitative studies |
| Population | Low and middle-income countries  Population subgroup of interest includes: adults/youths, children, women/girls, elderly, rural/urban and conflict regions | - Studies only on high income countries - Systematic reviews only on high income countries |
| Interventions | Studies with interventions that aim to improve social accountability and will include the following:   1. Access to information 2. Citizens’ voice and representation 3. Governance 4. Mobilisation actions 5. Resource monitoring actions |  |
| Outcomes | Studies that aim to improve social accountability as an outcome and include following:   1. Performance improvement of public and private actors/institutions 2. Service Quality 3. Allocative efficiency 4. Citizen Engagement 5. Inclusion 6. Responsiveness 7. Quality of life 8. Knowledge and Learning outcomes from the study |  |
| Study Quality | Low  Medium  High |  |
| Language | English | Exclude studies in other language |

## Table A4

##### **Screening tool**

| 1. | Is the paper published after 2000? | No | Exclude |
| --- | --- | --- | --- |
|  |  | Yes | Continue to q2 |
| 2. | Is the paper in English? | No | Exclude |
|  |  | Yes | Continue to q3 |
| 3 | Is the paper on low and middle-income countries? | No | Exclude |
|  |  | Yes | Continue to q4 |
|  |  |  |  |
| 4. | Is there any intervention about social accountability in the paper regarding access to information/ citizens’ voice/ governance/ mobilization/ resource allocation? | No | Exclude |
|  |  | Yes | Continue to q5 |
|  |  |  |  |
| 5. | Is the paper a quantitative evaluation (RCT/non-experimental with comparison group/ before-and-after design) or a systematic review? | No | Exclude |
|  |  | Yes | Include |
|  |  |  |  |
